# Pathogens, endosymbionts, and blood-meal sources of host-seeking ticks in the fast-changing Maasai Mara wildlife ecosystem

**DOI:** 10.1371/journal.pone.0228366

**Published:** 2020-08-31

**Authors:** Joseph Wang’ang’a Oundo, Jandouwe Villinger, Maamun Jeneby, George Ong’amo, Moses Yongo Otiende, Edward Edmond Makhulu, Ali Abdulahi Musa, Daniel Obado Ouso, Lillian Wambua

**Affiliations:** 1 International Centre of Insect Physiology and Ecology (*icipe*), Nairobi, Kenya; 2 School of Biological Sciences, University of Nairobi, Nairobi, Kenya; 3 Institute of Primate Research, National Museums of Kenya, Nairobi, Kenya; 4 Forensic and Genetics Laboratory, Kenya Wildlife Service, Nairobi, Kenya; 5 Department of Biochemistry and Molecular Biology, Egerton University, Egerton, Kenya; 6 Department of Medical Laboratory Sciences, Kenyatta University, Nairobi, Kenya; Tufts University Cummings School of Veterinary Medicine, UNITED STATES

## Abstract

The role of questing ticks in the epidemiology of tick-borne diseases in Kenya’s Maasai Mara National Reserve (MMNR), an ecosystem with intensified human-wildlife-livestock interactions, remains poorly understood. We surveyed the diversity of questing ticks, their blood-meal hosts, and tick-borne pathogens to understand potential effects on human and livestock health. By flagging and hand-picking from vegetation in 25 localities, we collected 1,465 host-seeking ticks, mostly *Rhipicephalus* and *Amblyomma* species identified by morphology and molecular analysis. We used PCR with high-resolution melting (HRM) analysis and sequencing to identify *Anaplasma*, *Babesia*, *Coxiella*, *Ehrlichia*, *Rickettsia*, and *Theileria* pathogens and blood-meal remnants in 231 tick pools. We detected blood-meals from humans, wildebeest, and African buffalo in *Rh*. *appendiculatus*, goat in *Rh*. *evertsi*, sheep in *Am*. *gemma*, and cattle in *Am*. *variegatum*. *Rickettsia africae* was detected in *Am*. *gemma* (MIR = 3.10) that had fed on sheep and in *Am*. *variegatum* (MIR = 250) that had fed on cattle. We found *Rickettsia* spp. in *Am*. *gemma* (MIR = 9.29) and *Rh*. *evertsi* (MIR = 200), *Anaplasma ovis* in *Rh*. *appendiculatus* (MIR = 0.89) and *Rh*. *evertsi* (MIR = 200), *Anaplasma bovis* in *Rh*. *appendiculatus* (MIR = 0.89), and *Theileria parva* in *Rh*. *appendiculatus* (MIR = 24). No *Babesia*, *Ehrlichia*, or *Coxiella* pathogens were detected. Unexpectedly, species-specific *Coxiella* sp. endosymbionts were detected in all tick genera (174/231 pools), which may affect tick physiology and vector competence. These findings show that ticks from the MMNR are infected with zoonotic *R*. *africae* and unclassified *Rickettsia* spp., demonstrating risk of African tick-bite fever and other spotted-fever group rickettsioses to locals and visitors. The protozoan pathogens identified may also pose risk to livestock production. The diverse vertebrate blood-meals of questing ticks in this ecosystem including humans, wildlife, and domestic animals, may amplify transmission of tick-borne zoonoses and livestock diseases.

## 1. Introduction

Wildlife ecosystems are known to be hotspots for a range of emerging diseases threatening human and livestock health [1–3]. The ecology of tick-borne pathogens (TBPs) is complex, often involving wildlife, domestic animals, and humans that not only provide blood-meals to maintain the tick populations but also serve as reservoirs and/or amplifiers of different TBPs [4, 5]. The majority of emerging pathogens are maintained asymptomatically by wildlife and are transmitted to humans and livestock by vectors such as ticks and mosquitoes. An upsurge of emerging tick-borne zoonoses has been witnessed globally in the recent decades, such as Lyme borreliosis, which affect humans in several developed countries [6], while the burden of the tick-borne diseases of livestock, such as East Coast fever (ECF), has persisted in sub-Saharan Africa [7]. Therefore, the study and control of TBPs demand a ‘One Health’ approach, requiring knowledge of the tick species, their host feeding preferences, habitat, and range [5].

The emergence and expansion of TBDs are increasingly linked to changes in the physical environment [8, 9]. It has been observed that ecosystems undergoing drastic changes (such as rapid vegetation cover degradation, changes in climate and land-use patterns) are likely to become “pathogenic landscapes” due to the increased connectivity and probability of contact between vectors and their animal and human hosts [10]. The Maasai Mara ecosystem in south-western Kenya represents one such fast-changing environment. While this ecosystem has been long recognized as a biodiversity hub and the home of the spectacular wildebeest migration termed “*the 7*^*th*^ *wonder of the world*”, it has faced severe threats and challenges in the last three decades driven by drastic changes in land use [11–13]. Significant land fragmentation has occurred in the Maasai Mara to accommodate an increasing number of conservancies, tourist lodges, human settlements, and agricultural developments [14]. Multiple land uses, such as pastoralism, commercial ranching, camping, tourism, and illegal grazing are being practiced concurrently, which favor the convergence of humans and domestic animals with wild animals and increase the risk of pathogen transmission [14–17]. We therefore aimed to understand the interactions between ticks, pathogens, and their vertebrate hosts in this fast-changing ecosystem. We based our study on questing ticks because this is the active stage of ixodid ticks seeking vertebrate hosts for blood-meals in which they pick up new pathogens and transmit them from previous blood-meals to new hosts, including humans and livestock.

## 2. Materials and methods

### 2.1. Study area

**Fig 1:**
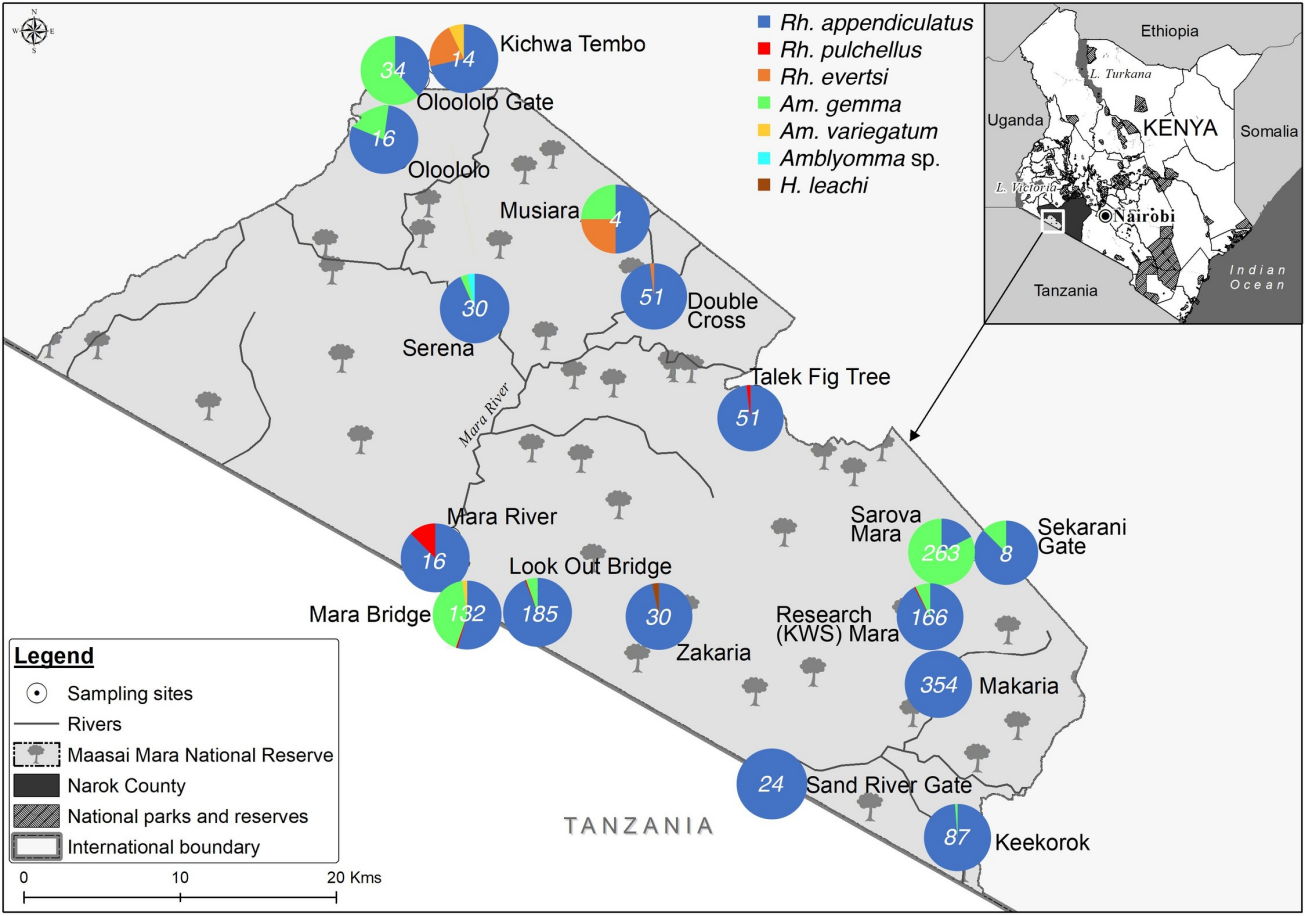
Distribution and abundance of tick species across various study sites of the Maasai Mara National Reserve. This map is Republished with data under CC BY licenses from the following sources: https://www.wri.org/resources/data-sets/kenya-gis-data from the World Institute Resources, 2007 [18]; https://africaopendata.org/dataset/kenya-counties-shapefile from openAfrica, 2015 [19]; and https://gadm.org/data.html from GADM, 2018 [20].

The MMNR lies within Narok County in southwestern Kenya (Fig 1) and is contiguous with the Serengeti National Park in northern Tanzania. The MMNR supports a high diversity of large and small mammals and is globally famous for the annual wildebeest migration involving 1.3 million wildebeest, 200,000 zebras, and hundreds of thousands of Thomson’s gazelles, topi, and elands [14]. This great migration into the Maasai Mara begins in July when these animals migrate from the Serengeti plains and end in October when they migrate back to the Serengeti. This annual migration event potentially introduces new tick-microbe associations, or more likely, promotes the mixing of strains from different areas. The MMNR also includes tourist lodges, hotels, conservancies and commercial ranches, as well as settlements inhabited by local indigenous Maasai tribe whose livelihoods are dependent on livestock, mainly cattle, goats and sheep. Sampling sites included areas in the wildlife reserve as well as those bordering homesteads, ranches and tourist hotels. Ethical clearance for this research in protected areas was sought from and approved by the Kenya Wildlife Service (KWS) Research Authorization committee.

### 2.2. Tick collection and identification

Questing ticks were collected from 25 sites in the MMNR during the great wildebeest migration in June–July of 2016 (Fig 1). Within each sampling site, we established survey plots measuring 100 m × 100 m comprising vertebrate resting areas, burrows, host routes and watering holes. Questing ticks were sampled within these plots between 10 am and 5 pm using a combination of flagging and picking from vegetation with gloved hands and a pair of forceps [21]. Flagging was carried out by slowly dragging a 1m^2^ white cotton cloth over the vegetation along 100-m transects. Ticks attached to the cloth were collected using forceps after each 10-m drag, put in sterile labeled tubes and frozen on dry ice while in the field, before being carried in liquid nitrogen to the lab at the International Centre of Insect Physiology and Ecology (*icipe*), Nairobi. Once in the lab, adult ticks were identified to genus and/or species level under a microscope (Stemi 2000-C, Zeiss, Oberkochen, Germany) based on morphological keys developed by Walker et al. [22]. Immature stages were identified to genus level against larvae and nymphs of conspecific adults of *Rhipicephalus* and *Amblyomma* laboratory colonies maintained in *icipe*’s tick unit and further confirmed by molecular analyses. To prevent contamination with exogenous DNA, sterile petri-dishes, gloves, forceps, and gloves were used while handling the ticks. After morphological identification, ticks were pooled according to the sampling site, species, and sex into groups of 1–11 adults, 1–20 nymphs, and 1–25 larvae.

### 2.3. DNA extraction from tick pools

Tick pools were homogenized in 1.5-ml Eppendorf tubes using zirconium oxide beads (Glen Mills, Clifton, NJ, USA) as previously described [23]. Genomic DNA was extracted from the homogenates using a protein precipitation method. Briefly, 300 μl of sterile cell lysis buffer (10 mM Tris-HCL [pH 8.0], 5 mM EDTA, 0.5% SDS) was added to the homogenate. The lysate was then incubated at 65°C for 60 minutes, followed by addition of 100 μl of protein precipitation solution (8 M ammonium acetate, 1 mM EDTA). The tubes were vortexed for 30 seconds and centrifuged for 10 min at 14,000 x g. The supernatants were aspirated and 300 μl of isopropanol was added to precipitate the DNA. Tubes were vortexed for 30 seconds, and the DNA was pelleted by centrifugation for 30 minutes at 14,000 x g. The DNA pellets were washed with 300 μl ice-cold 70% ethanol and air-dried for 12 hours before suspension in 50 μl of distilled deionized water. The quality and quantity of the extracted DNA samples were measured using a Nanodrop ND-1000 spectrophotometer (Thermo Scientific, Waltham, MA, USA) and diluted to 50 ng/μl for PCR. All DNA extracts were stored at -20°C until further use.

### 2.4. Molecular analyses of tick species

For molecular confirmation of species and genetic diversity, we selected representative immature and adult ticks of each tick species and amplified fragments of cytochrome oxidase 1 (CO1), 16S ribosomal ribonucleic acid (rRNA), and internal transcribed spacer 2 (ITS-2) gene markers from tick genomic DNA using PCR. The PCRs were performed in 10-μl reaction volumes that included 100 ng of DNA template, 1X HOT FIREPol® Blend Master Mix (Solis Biodyne, Estonia), 500 nM of each primer (S1 Table) and 5 μl PCR grade water. The following thermocycling conditions were used: Initial denaturation at 95°C for 15 min followed by 35 cycles of denaturation at 95°C for 20 s, annealing at 55°C (16S rRNA and CO1) and 65°C (ITS 2) for 30 s and extension at 72°C for 1 min, and a final extension at 72°C for 5 min. A no-template control with ddH_2_O in place of DNA was included in each run. PCR products were purified for sequencing using ExoSAP-IT Enzymatic PCR Product Clean-Up Kit (USB Corporation, Cleveland, OH, USA) according to the manufacturer’s instructions and sent to Macrogen (Netherlands) for capillary sequencing.

### 2.5. Screening of tick-borne pathogen DNA by PCR and High-Resolution Melting (PCR-HRM) analysis

A total of 231 tick pools were tested by PCR for the presence of pathogens belonging to the genera *Anaplasma*, *Coxiella*, *Ehrlichia*, *Rickettsia*, *Theileria*, and *Babesia* using genus-specific primers (S1 Table). The procedure entailed touch-down PCR amplification followed by melting of the amplicons in an HRM capable thermal cycler (Qiagen, Germany). The assays were performed in 10-μl reaction volumes, containing final concentration of 1x HOT FIREPol EvaGreen HRM mix (no ROX) (Solis BioDyne), 500 nM of the respective forward and reverse primers (S1 Table), 100 ng of template DNA and 5 μl PCR grade water. DNA samples of *Anaplasma phagocytophilum*, *Ehrlichia ruminantium*, and *R*. *africae* from an earlier study [24] were used as positive controls and no-template controls were also included. The touch-down PCR thermocycling conditions included an initial denaturation at 95°C for 15 min, followed by 10 cycles of amplification including denaturation at 94°C for 20 s; annealing for 25 s at 63.5–53.5°C (decreasing by 1°C per cycle), and 72°C for 30 s, followed by 25 cycles with an annealing temperature of 50.5°C and a final extension step at 72°C for 7 min. Cycling conditions described by Fard and Khalili [25] were used for *C*. *burnetii-*specific primers. An HRM step was thereafter performed in which amplicons were gradually melted from 75–90°C with 0.1°C increments every 2 s. Melting profiles were visualized within the Rotor-Gene Q Software v.2.1.0 (Build 9). Representative amplicons associated with each unique HRM profile were purified for sequencing.

### 2.6. Estimation of Minimum Infection Rate (MIR)

The minimum infection rate (MIR) of TBPs in each tick species in the MMNR was calculated using the formula: [number of pathogen positive tick pools / total number of ticks of that species tested] x 1000. The MIR assumes that only one tick is positive in a pool.

### 2.7. Analysis of tick blood-meal remnants by PCR-HRM

We investigated the vertebrate sources of tick blood-meals in all 231 tick pools, following established protocols [26, 27]. Briefly, genomic DNA from the tick pools was analyzed by PCR amplification of vertebrate cytochrome b (*cyt b*) and 16S ribosomal (r) RNA genes using primers listed in S1 Table. PCR reactions were set-up with similar reaction volumes and components as already described for pathogen PCR-HRM. DNA extracts from voucher wildlife specimens (obtained from the Kenya Wildlife Service) and livestock species listed here were included as positive controls: Blue wildebeest (*Connochaetes taurinus*), giraffe (*Giraffa camelopardalis*), impala (*Aepyceros melampus*), buffalo (*Syncerus caffer*), warthog (*Phacochoerus africanus*), Grant's gazelle (*Nanger granti*), hartebeest (*Alcelaphus buselaphus*), waterbuck (*Kobus ellipsiprymnus*), plain's zebra (*Equus quagga*), Kirk's dik-dik (*Madoqua kirkii*), Sable antelope (*Hippotagus niger*), lion (*Panthera leo*), cattle (*Bos taurus*), sheep (*Ovis aries*), and goat (*Capra hircus*). The amplicons with unique melt curves were purified for confirmation by Sanger sequencing.

### 2.8. Genetic and phylogenetic analyses

Using the MAFFT plugin in Geneious software version 11.1.4 (created by Biomatters) [28], all study nucleotide sequences were edited and aligned with related sequences identified by querying in the GenBank nr database using the Basic Local Alignment Search Tool (www.ncbi.nlm.nih.gov/BLAST/). The aligned DNA sequences were used to construct maximum likelihood phylogenetic trees using PHYML v.3.0 [29]. The phylogenies employed the Akaike information criterion [30] for automatic model selection and tree topologies were estimated using nearest neighbor interchange (NNI) improvements over 1,000 bootstrap replicates. Phylogenetic trees were visualized using FigTree v1.4.2.

## 3. Results

### 3.1. Tick species diversity

**Fig 2:**
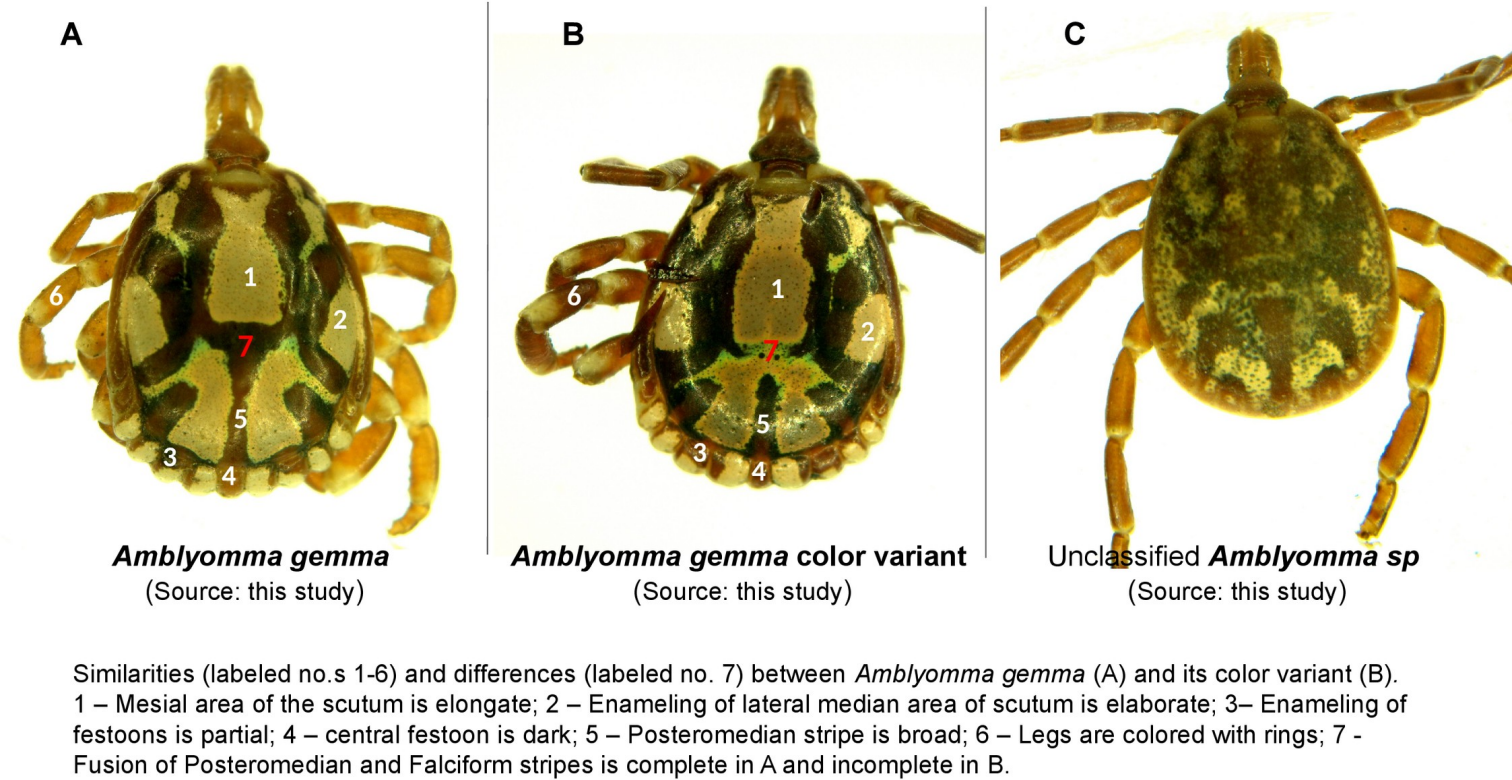
Comparison of selected *Amblyomma* species from this study: *Amblyomma gemma* and color variants of *Amblyomma gemma* ticks are depicted on panels A and B while an unclassified *Amblyomma* tick is shown in panel C. Similarities between *Amblyomma gemma* and color variants of *Amblyomma gemma* are labeled in white and differences in red colors respectively.

**Fig 3:**
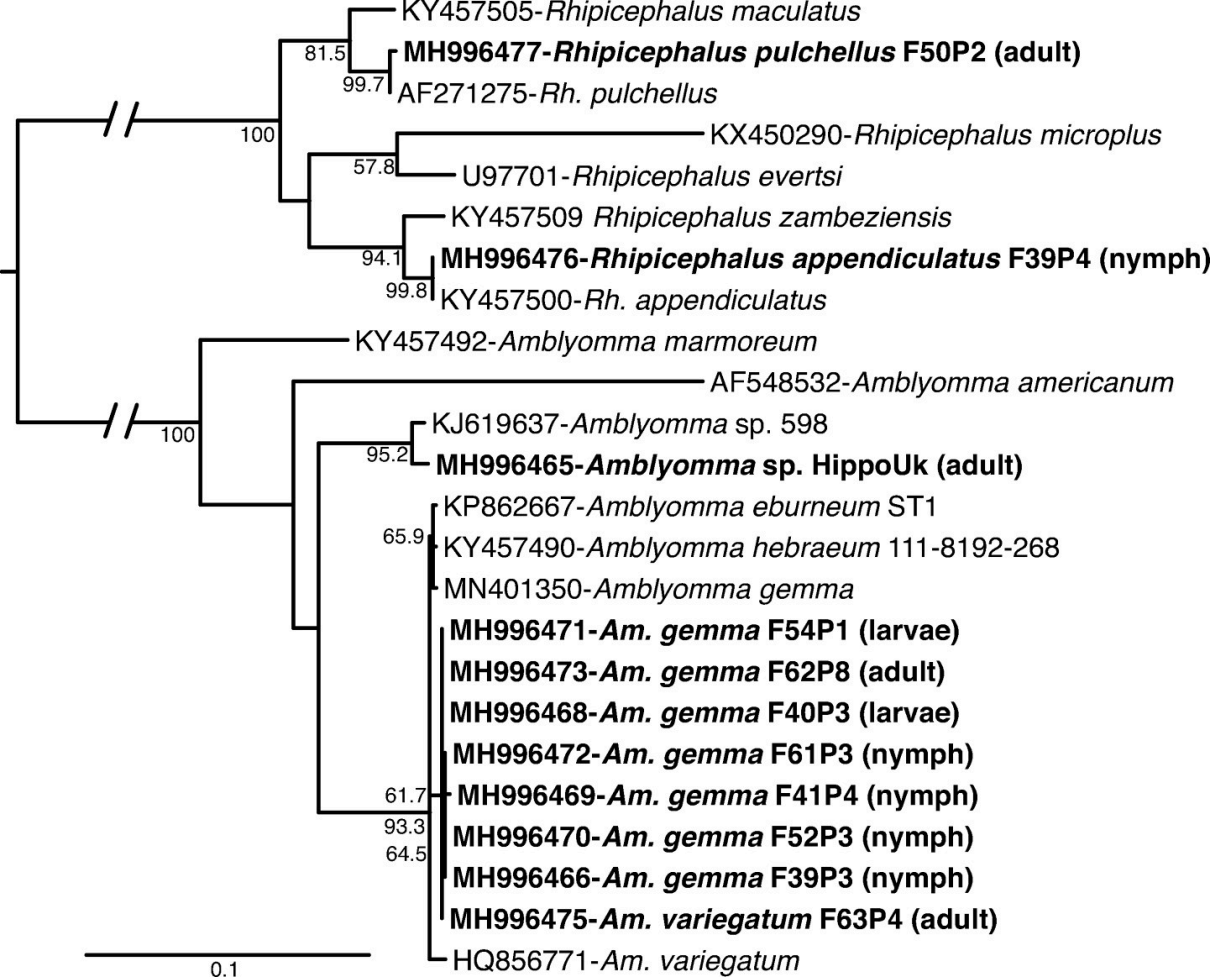
Maximum likelihood phylogenetic tree of tick ITS-2 gene sequences (739–1276 bp). GenBank accession numbers, species identifications, and isolates, and tick life stages (in brackets) are indicated for each sequence. Sequences from this study are indicated in bold letters. Bootstrap values at the major nodes are of percentage agreement among 1000 replicates. The branch length scale represents substitutions per site.

We collected a total of 1,465 questing ticks across the MMNR including 1137 adults, 97 nymphs, and 231 larvae. According to morphological and genetic analysis, *Rhipicephalus appendiculatus* comprised the highest proportion of the ticks sampled (n = 1125, 76.79%). Other species included *Rhipicephalus pulchellus* (n = 6, 0.41%), *Rhipicephalus evertsi* (n = 5, 0.34%) *Amblyomma gemma* (n = 323, 22.04%), *Amblyomma variegatum* (n = 4, 0.27%), *Amblyomma* sp. (n = 1, 0.07%) and *Haemaphysalis leachi* (n = 1, 0.07%) (Fig 1; Table 1). Additionally, we identified a proportion of *Am*. *gemma* ticks (n = 178/323), which displayed a subtle morphological variation, in that the joining of the posteromedian stripe to the falciform stripe was incomplete in the 178 individuals, relative to the reference images of *Am*. *gemma* in which the two stripes are fully joined in [22] (Fig 2). However, the ornamentation of their mesial and lateral median areas on the scutum was similar to that of *Am*. *gemma*, and they exhibited partial enameling of the festoons with the central festoon being dark as in *Am*. *gemma* [22] (Fig 2). Hence, for purposes of this work, these ticks were regarded as color variants of *Am*. *gemma*. Genetic analysis of CO1, 16S rRNA and ITS-2 sequences of both adult and immature individuals of these *Am*. *gemma* color variants was done, but only the ITS-2 marker yielded amplicons, with the sequences clustering together with ITS-2 sequences of *Am*. *gemma*, *Am*. *hebraeum*, *Amblyomma eburneum* and *Amblyomma variegatum* (Fig 3). One adult *Amblyomma* male tick which could not be morphologically identified to species level (Fig 2) but had an ITS-2 sequence with 99% identity to that of an *Amblyomma* sp. removed from the nostril of a traveler who had visited Lope National Park in Gabon [31] (Fig 3) was identified. Hence, for purposes of this work, this tick was termed as *Amblyomma* sp. (Fig 2). The ITS-2 sequences of eight *Rh*. *appendiculatus* nymphs were 100% identical to reference *Rh*. *appendiculatus* sequences (Fig 3).

**Table 1:** Abundance and diversity of tick species collected in this study.

Tick species	Number of ticks					Total No. of tick pools
	Larvae	Nymphs	Adult males	Adult females	Total No. (%)	
*Rhipicephalus appendiculatus*	30	8	470	617	1,125 (76.79%)	172
*Rhipicephalus pulchellus*	0	0	2	4	6 (0.41%)	5
*Rhipicephalus evertsi*	0	0	2	3	5 (0.34%)	4
*Amblyomma gemma*	201	89	21	12	323 (22.04%)	45
*Amblyomma variegatum*	0	0	1	3	4 (0.27%)	3
*Amblyomma* sp.	0	0	1	0	1 (0.07%)	1
*Haemaphysalis leachi*	0	0	0	1	1 (0.07%)	1
**Total**	**231**	**97**	**497**	**640**	**1,465**	**231**

### 3.2. Tick-borne pathogens identified

A total of 231 tick pools were screened for *Anaplasma*, *Babesia*, *Coxiella*, *Ehrlichia*, *Rickettsia*, and *Theileria* pathogen infections, based on the pooling strategy depicted in Table 1. The pathogens detected are summarized in Table 2. None of the pools showed any amplification for *Babesia* spp., *Ehrlichia* spp., or *Coxiella burnetii*.

**Table 2:** Tick-borne pathogens identified.

Tick-borne pathogen	Tick species	No. of tick pools assayed	Total No. of ticks	No. of positive pools	Minimum infection rate (MIR)
*Anaplasma bovis*	*Rh*. *appendiculatus*	172	1,125	1	0.89
*Anaplasma ovis*	*Rh*. *Evertsi*	4	5	1	200
	*Rh*. *appendiculatus*	172	1,125	1	0.89
*Rickettsia africae*	*Am*. *gemma*	45	323	1	3.10
	*Am*. *variegatum*	3	4	1	250
*Rickettsia* sp.	*Am*. *gemma*	45	323	3	9.29
*Rickettsia* sp.	*Rh*. *Evertsi*	4	5	1	200
*Theileria parva*	*Rh*. *appendiculatus*	172	1,125	27	24

**Fig 4:**
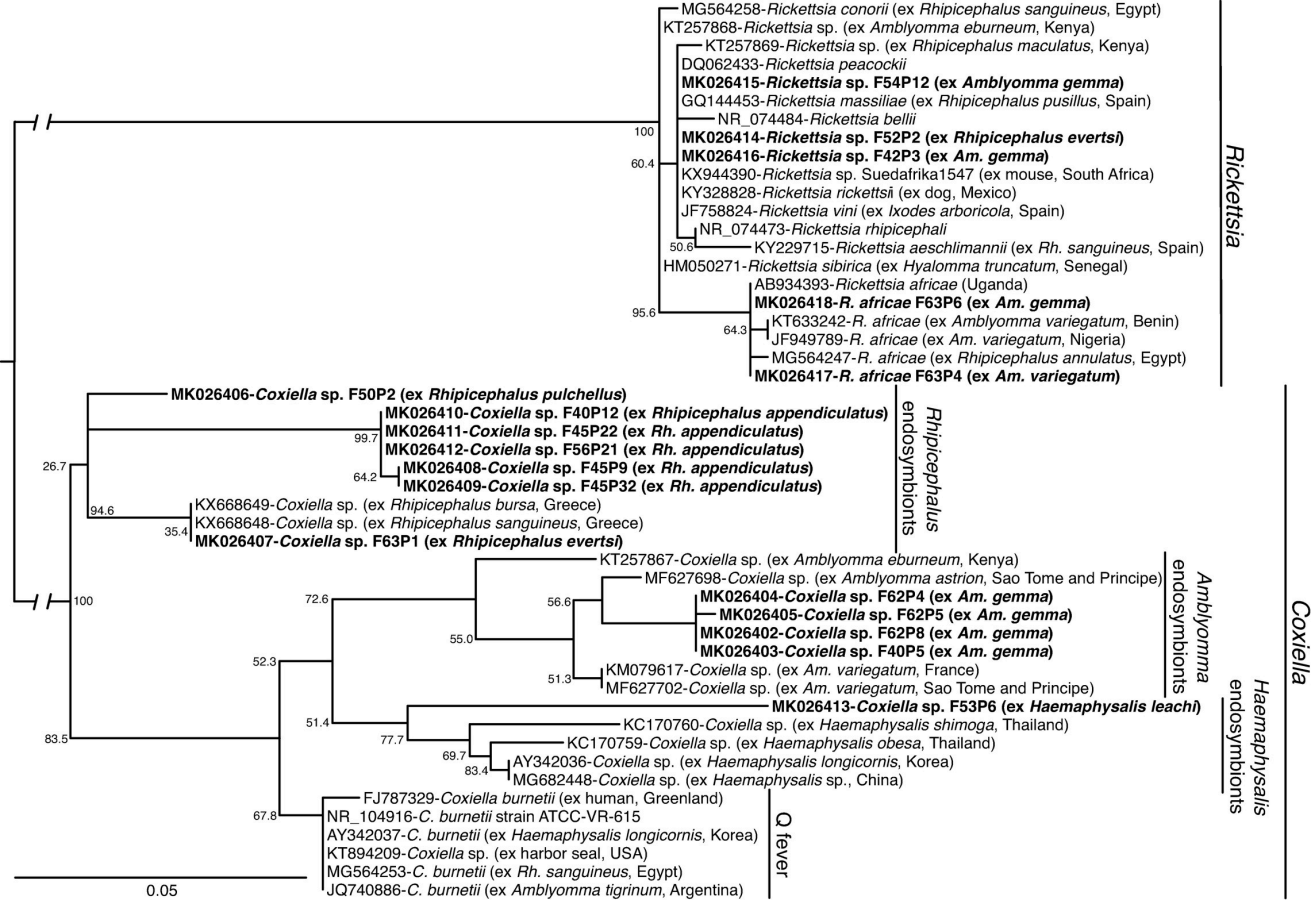
Maximum likelihood phylogenetic tree of *Rickettsia* and *Coxiella* 16S rRNA gene sequences (248–301 bp). GenBank accession numbers, species identifications, and isolates, with tick or vertebrate host species and country of origin in brackets, are indicated for each sequence. Sequences from this study are bolded. Bootstrap values at the major nodes are of percentage agreement among 1000 replicates. The branch length scale represents substitutions per site. The gaps indicated in the branches to the *Coxiella* and *Rickettsia* clades represent 0.10 substitutions per site.

Rickettsial DNA was amplified in six of the tick pools. Of these, *Rickettsia africae* 16S rDNA sequences sharing 100% identity with an isolate from Uganda were detected in two tick pools comprising of one adult *Am*. *gemma* female (MIR = 3.10) and one adult *Am*. *variegatum* male (MIR = 250), both from the Kichwa Tembo area of the MNNR (Fig 4; Table 2; S2 Table). Additionally, the DNA of unclassified *Rickettsia* spp. was amplified from one *Rh*. *evertsi* female tick (MIR = 200) and three *Am*. *gemma* (two males and one female; MIR = 9.29) ticks. All but one of these rickettsial 16S rDNA sequences were 99–100% identical to *Rickettsia* sp. Suedafrika1547 (GenBank accession KX944390) and clustered with other spotted fever groups (SFG) rickettsiae, including *Rickettsia massiliae*, *Rickettsia rhipicephali*, *Rickettsia amblyommii*, and *Rickettsia raoultii* based on maximum likelihood phylogenetic analysis of a partial sequence of the 16S rRNA gene (Fig 4). On further attempts to classify the *Rickettsia* spp. to the species level using the *ompB* gene, amplicons from two pools of *Am*. *gemma* similarly showed 97% identity to *Rickettsia aeschlimannii*, *R*. *rhipicephali*, *R*. *massiliae*, and *R*. *raoultii* (GenBank accessions MF002557, CP013133, KT835123, FN651773, respectively).

*Anaplasma* spp. 16S rDNA was amplified in two pools of *Rh*. *appendiculatus* and one pool of *Rh*. *evertsi* adult ticks (Table 2). The *Anaplasma* 16S rDNA sequences in one of the *Rh*. *appendiculatus* adult pools (comprising of seven male ticks from Double Cross area; MIR = 0.89) was 100% identical to reference *Anaplasma bovis* (S2 Table). The *Anaplasma* 16S rRNA sequences from the other *Rh*. *appendiculatus* adult pool (comprising of seven female ticks sampled from Look Out Bridge area; MIR = 0.89) and the *Rh*. *evertsi* adult pool (comprising of a female tick sampled from Kichwa Tembo area; MIR = 200) were 100% identical to reference *Anaplasma ovis* (S2 Table).

*Theileria* spp. 18S rRNA sequences were amplified from 27 out of 172 pools of *Rh*. *appendiculatus* adult ticks (MIR = 24) collected from Makaria, Sarova Mara, Mara Bridge and Oloololo Gate regions of the MMNR (Table 2; S2 Table). Sequences from representative samples of positive pools were 100% identical to *T*. *parva* isolates detected in Zambia, Kenya, Uganda, and Tanzania (S2 Table).

### 3.3. Tick-borne endosymbionts identified

A total of 174/231 pools showed positive amplification for *Rickettsia* 16S rRNA but were negative for the *Rickettsia ompB* gene. Upon further sequencing and analysis, the amplicons revealed the presence of *Coxiella* sp. endosymbionts. These tick pools with *Coxiella* sp. endosymbionts yielded no amplification when screened with *C*. *burnetii*-specific primers. *Coxiella* sp. endosymbionts were detected in 80.23% of *Rh*. *appendiculatus* pools (138/172), 100% of *Rh*. *pulchellus* pools (5/5), 25% of the *Rh*. *evertsi* pools (1/4), 60% of *Am*. *gemma* pools (27/45), 66.67% *Am*. *variegatum* pools (2/3), and in the single *H*. *leachi* pool. The endosymbionts were only absent in the single *Amblyomma* sp. tick reported in section 3.1 above. The *Coxiella* sp. endosymbionts were distributed across all sites in the MMNR (S2 Table). Phylogenetic analysis demonstrated that these endosymbionts clustered in three host-specific clades associated with *Amblyomma*, *Rhipicephalus*, and *Haemaphysalis* ticks (Fig 4).

We further analyzed the tick pools with *Coxiella* sp. endosymbionts for co-infection with pathogens. The results showed that two pools of adult *Rh*. *appendiculatus* were co-infected with the aforementioned *A*. *ovis* and *A*. *bovis* (section 3.2). Additionally, of the 27 *Rh*. *appendiculatus* tick pools that were infected with *T*. *parva*, 70% (19/27) were co-infected with *Coxiella* sp. endosymbionts.

### 3.4. Vertebrate blood-meal sources in questing ticks

A total of 231 tick pools were tested by PCR for vertebrate sources of blood-meals. From these, only nine tick pools, all of which comprised of single individuals, had identifiable blood-meals (Table 3; S1 Fig). No vertebrate blood-meal source was detected in the remaining 222 tick pools. Analysis of vertebrate *cyt b* sequences in these ticks revealed human blood-meals in one *Rh*. *appendiculatus* tick, and one *Am*. *gemma* tick. Blue wildebeest (*Connochaetes taurinus*) blood-meals were detected in two *Rh*. *appendiculatus* ticks, while African buffalo (*Syncerus caffer*) blood-meal was detected in one *Rh*. *appendiculatus* tick. We also detected goat (*Capra hircus*) blood-meal in one *Rh*. *evertsi* tick. Amongst *Amblyomma* ticks, two *Am*. *gemma* ticks had blood-meals from sheep (*Ovis* sp.), while one *Am*. *variegatum* had a blood-meal from cattle (*Bos taurus*) (Table 3; S1 Fig). The aforementioned *Rickettsia africae* infections (section 3.2) were detected in one of the *Am*. *gemma* ticks with a blood-meal from sheep and one of the *Am*. *variegatum* with a blood-meal from cattle.

**Table 3:** Summary of vertebrate sources of blood-meals in individual adult ticks detected by PCR and sequencing in questing ticks collected in this study.

Host tick species	No. of tick pools assayed	No. of pools positive for blood-meal *	Vertebrate source of blood-meal	TBP detected	Sampling location	Submitted GenBank accessions
*Rhipicephalus evertsi*	4	1	Goat (*Capra hircus*)	ND	Kichwa Tembo	MH997915
*Rhipicephalus appendiculatus*	172	2	Blue wildebeest (*Connochaetes taurinus*)	ND	Sand River Gate and Zakaria	MH997918, MH997919
		1	African buffalo (*Syncerus caffer*)	ND	Kichwa Tembo	MH997914
		1	Human (*Homo sapiens*)	ND	Oloololo Gate	MH997917
*Amblyomma gemma*	45	1	Human (*H*. *sapiens*)	ND	Oloololo Gate	MH997916
		2	Sheep (*Ovis sp*.)	*Rickettsia africae*	Kichwa Tembo	S1 Fig
*Amblyomma variegatum*	3	1	Cattle (*Bos taurus*)	*Rickettsia africae*	Kichwa Tembo	S1 Fig
*Rhipicephalus pulchellus*	5	0	ND	ND	ND	-
*Amblyomma* sp.	1	0	ND	ND	ND	-
*Haemaphysalis leachi*	1	0	ND	ND	ND	-

*—these pools comprised of single individuals; **TBP–**Tick-borne pathogen; ND–not detected.

## 4. Discussion

Concerns around the role of wildlife ecosystems as hotspots for a range of emerging diseases threatening human and livestock health have been rising, especially in areas where free-ranging wild animals regularly interact with domestic livestock and humans [1–3]. This study provides molecular evidence of the presence of the zoonotic *R*. *africae*, uncharacterized *Rickettsia* spp. and veterinary pathogens (including *A*. *bovis*, *A*. *ovis*, and *T*. *parva*) in questing ticks collected from the MMNR. We also report that questing ticks in this wildlife ecosystem feed on humans, wildlife, and domestic animals. Additionally, we report the presence of diverse species-specific *Coxiella* sp. endosymbionts in questing ticks in the MMNR. These findings are important to public and veterinary health strategies mitigating possible disease outbreaks in this fast-changing wildlife ecosystem in eastern Africa.

Diverse species of ticks were identified in this study, including *Amblyomma*, *Rhipicephalus*, and *Haemaphysalis* genera, which have previously been reported to occur in the Maasai Mara region [32, 33]. A notable finding of this study was the identification of color variants of *Am*. *gemma* which were widely distributed across most study sites in this ecosystem, albeit not reported in previous studies in this locale or other regions of Kenya. This morphological variation in *Am*. *gemma* has been reported by Walker and co-workers [22], where a minority of populations may exhibit incomplete fusion of the posteromedial and falciform stripes, as is evident in this study. Intraspecific morphological variations are also known to occur in other *Amblyomma* species with studies elsewhere demonstrating phenotypic plasticity within this genus [34, 35]. These findings highlight the important of utmost care in morphological identification of ticks and the need for concurrent genetic studies with nuclear and mitochondrial markers.

Analysis of tick-borne zoonotic pathogens in this study corroborate findings of previous studies in Kenya, which have demonstrated the circulation of zoonotic SFG *Rickettsia* in ticks from various ecologies [24, 36, 37]. The presence of *R*. *africae*, which causes a potentially fatal, but as yet neglected febrile illness, was first reported in this ecosystem in 2003 [32]. Since then, this pathogen has been highlighted as a threat to international travelers and local communities, yet in clinical settings in Kenya, no routine diagnosis for the pathogen in humans is done [38–43]. In this study, *R*. *africae* was detected in *Am*. *gemma* and *Am*. *variegatum* ticks, confirming the strong link between ticks of *Amblyomma* species and the epidemiology of *R*. *africae* in sub-Saharan Africa [37, 38, 44]. Further, *R*. *africae*-infected ticks were sampled from the Kichwa Tembo area in the MMNR, which is a wildlife-human interface dominated by tented camps and resorts. This may present a health hazard to local and international tourists visiting the reserve, further underpinning the need for continuous xenosurveillance and for clinicians in the Maasai Mara region and other wildlife ecosystems to include SFG rickettsiosis in the differential diagnosis of febrile cases. Nevertheless, knowledge gaps on SGF rickettsiosis in Kenya persist, including the level of risk posed to humans by infected ticks, epidemiological evidence of the disease in human populations, and factors underlying infection and transmission dynamics of the pathogens by ticks and other vectors.

Unclassified *Rickettsia* spp. of unknown pathogenicity were detected in *Rh*. *evertsi* and *Am*. *gemma* ticks. Previous reports have also demonstrated a high diversity of *Rickettsia* spp. in Kenya [24, 37, 45]. Although we cannot associate them with human illness, the potential pathogenicity of these rickettsiae cannot be overlooked, as many tick-isolated *Rickettsia* initially characterized as non-pathogenic are now recognized as pathogens [46]. However, given the increasing reports of novel species and uncharacterized variants of unknown pathogenicity in Kenya, it will be important to undertake further characterization of the unclassified *Rickettsia* spp. from this study, using a combination of genetic markers such as *ompA*, *gltA*, *sca4*, 17kDa in addition to the *ompB* gene that was utilized in the present study.

This study also found evidence of livestock pathogens in the ticks surveyed, key among which were *T*. *parva*, *A*. *bovis*, and *A*. *ovis*. *Theileria parva* was the most prevalent livestock pathogen and was detected solely in *Rh*. *appendiculatus*. *Theileria parva* causes ECF, which remains the most economically important parasitic disease in cattle in eastern and southern Africa [22, 47]. Therefore, our findings confirm that the distribution of *T*. *parva* is closely associated with *Rh*. *appendiculatus* ticks [47]. These findings may imply that a similar control approach using the Infection and Treatment Method (ITM) with the live vaccine termed as the “*Muguga cocktail*” [48] can be applied in this setting in MNNR to control potential livestock losses from ECF. However, it has been demonstrated that proximity to buffaloes, the natural reservoir of *T*. *parva*, is associated with high *T*. *parva* diversity [49], which was not discernible from the current study. Nonetheless, the high prevalence of *T*. *parva* detected in *Rh*. *appendiculatus* ticks sampled in this study indicate the need to comprehensively analyze the genotypes of *T*. *parva* isolates from ticks and cattle in this ecosystem, relative to the proximity to buffalo niches and vaccination status.

*Coxiella burnetii* was not detected in this study, augmenting prior findings of Ndeereh and co-workers [33], who also reported the absence of *C*. *burnetii* in both ticks and animals in this ecosystem. However, tick species-specific *Coxiella* sp. endosymbionts were identified in 75.3% of adult ticks across the three genera sampled in this study, by sequencing amplicons obtained using the 16S rRNA primers for *Rickettsia* species described by Nijhof and co-workers [50]. Our results therefore suggest that these primers may have potential applications in the study of *Coxiella* sp. endosymbionts in ticks, as none of the respective samples yielded positive PCR results when screened with *C*. *burnetii*-specific primers. While the current findings are interesting, there is a need for subsequent studies to further characterize the *Coxiella* endosymbionts using housekeeping genes such as *fusA*, *rpsF*, and *rpsG* in addition to the 16S rRNA [51]. Previous investigations using the same assays as in this study, sequenced a *Coxiella* sp. endosymbiont from an *Am*. *eburneum* nymph, but not from other tick samples from the coastal region of Kenya [24] or from the Lake Victoria region [52], suggesting that the observed prevalence in the current study may due to stable inheritance of the endosymbionts within local lineages of ticks. Their distinct association with particular species of ticks as shown in this study, prompts further investigations to establish whether or not the *Coxiella* sp. endosymbionts have potential roles in vector nutrition, reproductive fitness and vector competence of ticks in this ecosystem [53, 54]. These findings also underpin the fact that PCR results of *Coxiella* infection in ticks must be interpreted with caution, especially if the amplified DNA products are not sequenced [55–57].

The identification of blood-meal remnants in questing ticks from across the MMNR has lent insight into the feeding behavior of ticks and the risk of tick-borne diseases in this area. Although we amplified blood-meal remnants in only nine of the sampled adult questing ticks, diverse sources of vertebrate sources including goat, sheep, blue wildebeest, African buffalo, cattle, and humans were identified, indicating that the host-seeking patterns of ticks in the MMNR were dynamic. The host blood-meal analysis was poorly sensitive as only 3.9% of 231 tick pools yielded an identifiable blood-meal. This may be because questing ticks can have their last blood-meal from their previous life stage up to one year before collection, which may have been degraded by digestive and hemolytic processes in the tick midgut [58, 59]. Nevertheless, our finding that *Rh*. *appendiculatus* ticks had fed on humans, blue wildebeest, and African buffalo, not only confirms previous knowledge that *Rh*. *appendiculatus* infests a wide range of Bovidae [22, 47], but also suggest a more diverse host-seeking pattern in involving humans. Although the known domestic hosts of adult *Rh*. *evertsi* are horses, donkeys, cattle, and sheep [22], we also detected a remnant blood-meal from a goat. We also found that *Am*. *variegatum* from the MMNR had fed on cattle, which is consistent with the previous report that all stages of this tick infest cattle, sheep, and goats [22]. The findings of blood-meals from domestic animals in a wildlife park setting also confirm the problem of encroachment of this wildlife ecosystem by humans and their livestock. The color variants of *Am*. *gemma* ticks identified in this study had fed on humans and although no pathogens were identified in this species, it nevertheless highlights the species as an important human parasite. We also detected *R*. *africae* infection in one *Am*. *gemma* that had fed on sheep and in one *Am*. *variegatum* that had fed on cattle. This suggests that sheep and cattle may be important in the epidemiology of African tick bite fever in the MMNR.

## 5. Conclusions

This study provides insights into the diversity of ticks, their microbes, and their blood-meal sources in the Maasai Mara ecosystem in Kenya, which is marked by rapid changes in land-use and considerable encroachment by humans and livestock. We report the observation of color variants of *Amblyomma gemma*, and also demonstrate the presence and possible circulation of etiological agents of spotted group rickettsiosis that may pose serious constraints to human health in the MMNR, as well as anaplasmosis, and theileriosis that may impede livestock production. The results also highlight that tick species in the MMNR feed on humans as well as diverse wildlife and livestock, including blue wildebeest, African buffalo, goat, sheep, and cattle. Our data also show that ticks from the MMNR harbor tick species-specific *Coxiella* spp. endosymbionts, highlighting the need for further studies to understand the role of these endosymbionts in tick physiology and vector competence.

## Supporting information

S1 Fig Alignment of short vertebrate 16S rRNA sequences of goat, sheep, and cow blood-meals amplified from ticks in this study. Sequences from this study are highlighted in (bold) against closest sequences available in GenBank. Color code of nucleotides are depicted as Green = Thymine; Red = Adenine; Blue = Cytosine; Yellow = Guanine.
(TIFF)

S1 Table PCR primer pairs used in this study. (DOCX)

S2 Table Detailed summary of geographical sources, sequence identities, and GenBank accessions of tick-borne pathogens and endosymbionts detected in this study. (DOCX)

## Abbreviations

cyt b: cytochrome b
ECF: East Coast fever
HRM: high-resolution melting
icipe: International Centre of Insect Physiology and Ecology
ITS-2: internal transcribed spacer 2
rRNA: ribosomal RNA
nt: nucleotide
MMNR: Maasai Mara National Reserve
MIR: minimum infection rate
ML-EID: Martin Lüscher Emerging Infectious Diseases (Laboratory)
SFG: Spotted fever group (rickettsioses)
TBP: tick-borne pathogen
TBD: tick-borne disease
