# Supplementary figures and images for: Pathogens, endosymbionts, and blood-meal sources of host-seeking ticks in the fast-changing Maasai Mara wildlife ecosystem
Source: PLoS One. 2020 Aug 31;15(8):e0228366. doi: 10.1371/journal.pone.0228366 (PMC7458302; doi:10.1371/journal.pone.0228366)

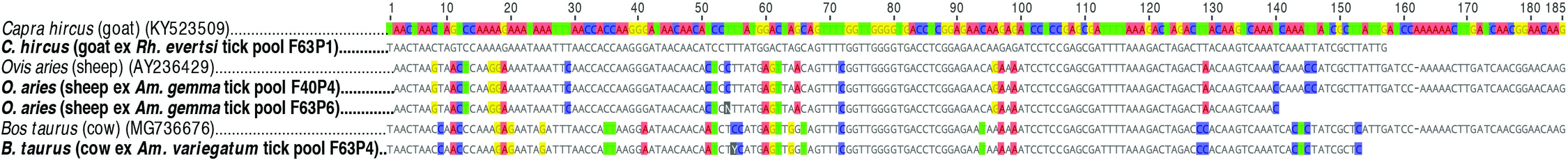

Supplement: S1 Fig — Sequences from this study are highlighted in (bold) against closest sequences available in GenBank. Color code of nucleotides are depicted as Green = Thymine; Red = Adenine; Blue = Cytosine; Yellow = Guanine. (TIFF) [file pone.0228366.s001.tiff]
