# Supplementary material for: Pathogens, endosymbionts, and blood-meal sources of host-seeking ticks in the fast-changing Maasai Mara wildlife ecosystem
Source: PLoS One. 2020 Aug 31;15(8):e0228366. doi: 10.1371/journal.pone.0228366 (PMC7458302; doi:10.1371/journal.pone.0228366)
Supplement: S1 Table — (DOCX) [file pone.0228366.s002.docx]

|  | **Target gene** | **Primer name** | **Primer sequence (5’-3’)** | **Amplicon**  **Size (bp)** | **Reference** |
| --- | --- | --- | --- | --- | --- |
| **Tick species** | 16S rRNA | F-RMI16S  R-RMI16S | AATTGCTGTAGTATTTTGAC  TCTGAACTCAGATCAAGTAG | 450 | [1] |
|  | CO1 | Lep R1  Lep F1 | ATTCAACCAATCATAAAGATATTGG  TAAACTTCTGGATGTCCAAAAAATCA | 658 | [2] |
|  | ITS2 | TITS2F1  TITS2R1 | CGAGACTTGGTGTGAATTGCA  TCCCATACACCACATTTCCCG | 750-2000 | [3] |
| ***Rickettsia* spp.** | 16S rRNA | Rick-F1  Rick-R2 | GAACGCTATCGGTATGCTTAACACA  CATCACTCACTCGGTATTGCTGGA | 364 | [4] |
|  | ompB | ompB 2788  ompB 3599 | AAACAATAATCAAGGTACTGT  TACTTCCGGTTACAGCAAAGT | 856 | [5] |
| ***Ehrlichia* spp.** | 16S rRNA | *Ehr* 16S F  *Ehr* 16S R | CGTAAAGGGCACGTAGGTGGACTA  CACCTCAGTGTCAGTATCGAACCA | 200 | [6] |
|  |  | *Ehr*JV F  *Ehr*JV R | GCAACCCTCATCCTTAGTTACCA  TGTTACGACTTCACCCTAGTCAC | 300 | [7] |
| ***Anaplasma* spp.** | 16S rRNA | *Ana* 16S F  *Ana* 16S R | GGGCATGTAGGCGGTTCGGT  TCAGCGTCAGTACCGGACCA | 112-200 | [6] |
|  |  | *Ana* JV F  *Ana* JV R | CGGTGGAGCATGTGGTTTAATTC  CGRCGTTGCAACCTATTGTAGTC | 300 | [7] |
| ***Theileria and Babesia* spp.** | 18S rRNA | RLB-F  RLB-R | GAGGTAGTGACAAGAAATAACAATA  TCTTCGATCCCCTAACTTTC | 450 | [8] |
| ***Coxiella burnetii*** | IS1111 | Trans 1  Trans 2 | TATGTATCCACCGTAGCCAGTC  CCCAACAACACCTCCTTATTC | 687 | [9] |
| **Vertebrate bloodmeals** | *cyt b* | Cytb For  Cytb Rev | CCCCTCAGAATGATATTTGTCCTCA  CATCCAACATCTCAGCATGATGAAA | 383 | [10] |
|  | 16S rRNA | Vert 16S For  Vert 16S Rev | GAGAAGACCCTRTGGARCTT  CGCTGTTATCCCTAGGGTA | 200 | [11] |

**S1 Table. PCR primer pairs used in this study**

**References (Sources of PCR primers)**

1. Brahma RK, Dixit V, Sangwan AK, Doley R. Identification and characterization of *Rhipicephalus (Boophilus) microplus* and *Haemaphysalis bispinosa* ticks (Acari: Ixodidae) of North East India by ITS2 and 16S rDNA sequences and morphological analysis. Exp Appl Acarol. 2014;62(2):253–65.

2. Hebert PDN, Penton EH, Burns JM, Janzen DH, Hallwachs W. Ten species in one: DNA barcoding reveals cryptic species in the neotropical skipper butterfly *Astraptes fulgerator*. Proc Natl Acad Sci U S A. 2004;101(41):14812–7.

3. Chitimia L, Lin RQ, Cosoroaba I, Braila P, Song HQ, Zhu XQ. Molecular characterization of hard and soft ticks from Romania by sequences of the internal transcribed spacers of ribosomal DNA. Parasitol Res. 2009;105(4):907–11.

4. Nijhof AM, Bodaan C, Postigo M, Nieuwenhuijs H, Opsteegh M, Franssen L, et al. Ticks and associated pathogens collected from domestic animals in the Netherlands. Vector-Borne and Zoonotic Diseases. 2007;7(4):585–596.

5. Roux V, Raoult D. Phylogenetic analysis of members of the genus *Rickettsia* using the gene encoding the outer-membrane protein rOmpB (ompB). Int J Syst Evol Microbiol. 2000;50(4):1449–55.

6. Tokarz R, Kapoor V, Samuel JE, Bouyer DH, Briese T, Lipkin WI. Detection of tick-borne pathogens by masstag polymerase chain reaction. Vector-Borne Zoonotic Dis. 2009;9(2):147–51.

7. Mwamuye MM, Kariuki E, Omondi D, Kabii J, Odongo D, Masiga D, et al. Novel *Rickettsia* and emergent tick-borne pathogens: A molecular survey of ticks and tick-borne pathogens in Shimba Hills National Reserve, Kenya. Ticks and Tick-borne Diseases. 2017 Feb;8(2):208–218.

8. Georges K, Loria GR, Riili S, Greco A, Caracappa S, Jongejan F, et al. Detection of haemoparasites in cattle by reverse line blot hybridisation with a note on the distribution of ticks in Sicily. Vet Parasitol. 2001;99(4):273–86.

9. Hoover TA, Vodkin MH, Williams JC. A *Coxiella burnetii* repeated DNA element resembling a bacterial insertion sequence. J Bacteriol. 1992;174(17):5540–8.

10. Boakye DA, Tang J, Truc P, Merriweather A, Unnasch TR. Identification of bloodmeals in haematophagous Diptera by cytochrome B heteroduplex analysis. Med Vet Entomol. 1999;13(3):282–7.

11. Omondi D, Masiga DK, Ajamma YU, Fielding BC, Njoroge L, Villinger J. Unraveling host-vector-arbovirus interactions by two-gene high resolution melting mosquito bloodmeal analysis in a Kenyan wildlife-livestock interface. PLoS One. 2015;10(7):e0134375.
