# Supplementary material for: Pathogens, endosymbionts, and blood-meal sources of host-seeking ticks in the fast-changing Maasai Mara wildlife ecosystem
Source: PLoS One. 2020 Aug 31;15(8):e0228366. doi: 10.1371/journal.pone.0228366 (PMC7458302; doi:10.1371/journal.pone.0228366)
Supplement: S2 Table — (DOCX) [file pone.0228366.s003.docx]

**S2 Table: Detailed summary of geographical sources and sequence identities and Genbank accessions of tick-borne pathogens and endosymbionts detected in this study**

| **Sampling locations** | **Locus** | **Life cycle stage** | **Tick species with the pathogen** | **Closest BLAST hit, Reference accession** | **Sequence identity**  **(%)** | **Submitted GenBank accession** |
| --- | --- | --- | --- | --- | --- | --- |
| Kichwa Tembo | 16S rRNA | Adult | *Rh. evertsi* | *A. ovis,* KX579073 | 100 | MK026419 |
| Look Out Bridge | 16S rRNA | Adult | *Rh. appendiculatus* | *A. ovis,* KX579073 | 100 | MK026421 |
| Double Cross | 16S rRNA | Adult | *Rh. appendiculatus* | *A. bovis,* U03775 | 100 | MK026420 |
| Kichwa Tembo | 16S rRNA | Adult | *Am. variegatum* | *R. africae,* AB934393 | 100 | MK026417 |
| Kichwa Tembo | 16S rRNA | Adult | *Am. gemma* | *R. africae,* AB934393 | 100 | MK026418 |
| Kenya Wildlife Services Research Stations (KWS) Mara | 16S rRNA | Adult | *Am. gemma* | *Rickettsia* sp. Suedafrika1547, KX944390 | 99 | MK026415 |
| Mara Bridge | 16S rRNA | Adult | *Am. gemma* | *Rickettsia* sp. Suedafrika1547, KX944390 | 100 | MK026416 |
| Kenya Wildlife Services Research Stations (KWS) Mara | ompB | Adult | *Am. gemma* | *Rickettsia sp.,* KT835128  *R. aeschlimannii,* MF002557  *R. rhipicephali,* CP013133  *R. massiliae,* KT835123  *R. raoultii,* FN651773 | 97 | MH997912 - MH997913 |
| Makaria;  Sarova Mara,  Mara Bridge,  Oloololo Gate | 18S rRNA | Adult | *Rh. appendiculatus* | *T. parva,* MG952922*,* MG952923, MG952924, MG952926, | 98-100 | MH929321-MH929324 |
| All sampling sites | 16S rRNA | Adult | *Rh. appendiculatus,*  *Rh. pulchellus,*  *Rh. evertsi,*  *Am. gemma,*  *Am* cf. *gemma,*  *Am. variegatum,*  *H. leachi* | *Coxiella* sp. endosymbiont, D84559,  KT257867 | 96-100 | MK026402-MK026413 |
